# The Correlation between PSCA Expression and Neuroendocrine Differentiation in Prostate Cancer

**DOI:** 10.1155/2020/5395312

**Published:** 2020-09-24

**Authors:** Qian Xiang, Zhiguo Zhu, Lianmin Luo, Jiamin Wang, Yangzhou Liu, Yihan Deng, Mingda Zhou, Zhigang Zhao

**Affiliations:** Department of Urology & Andrology, Minimally Invasive Surgery Center, Guangdong Provincial Key Laboratory of Urology, The First Affiliated Hospital of Guangzhou Medical University, Guangzhou, Guangdong, China

## Abstract

The prostate stem cell antigen (PSCA), as a predominantly prostate-specific marker, is overexpressed in most prostate cancer specimens, is positively correlated with prostate cancer androgen independence, and has the potential to be treated with castration-resistant prostate cancer (CRPC) as a gene therapy target. Using the typical androgen deprivation therapy, most tumors will progress to CRPC, as well as develop into neuroendocrine prostate cancer (NEPC) characterized by the expression of neuroendocrine markers such as enolase 2 (NSE). Our study was aimed at investigating the expressions of PSCA and NSE and the relationship between the two markers, as well as the correlation between the PSCA and NSE expressions and the clinicopathological parameters in prostate cancer specimens from 118 patients by using immunohistochemistry. Our results demonstrated that the PSCA and NSE protein expressions did not correlate with the prostate cancer patients' age or the hormone therapy but showed a significant correlation with the pathological tumor stage of prostate cancer, the Gleason score, and the presence of metastasis. There is a positive association between PSCA and NSE but a negative one between the prostate-specific antigen (PSA) and PSCA or between PSA and NSE. High PSCA and NSE expressions correlated with a poor prognosis in prostate cancer patients. PSCA may play an important role in the progression of neuroendocrine prostate cancer (NEPC).

## 1. Introduction

Prostate cancer is the most commonly diagnosed malignancy and remains the second leading cause of cancer-related death in American men [1]. Its incidence is rising rapidly with the popularization of the prostate-specific antigen- (PSA-) based screening for prostate cancer in China. Although some great improvements can be seen in the diagnosis and treatment of the disease, the outcome of patients with advanced prostate cancer is still not getting better [2].

The prostate stem cell antigen (PSCA), a 123-amino acid glycoprotein belonging to the Thy-1/Ly-6 family of glycosylphosphatidylinositol- (GPI-) anchored cell surface antigens, is a predominantly prostate-specific marker [3]. As a result, it has been reported that PSCA is overexpressed in most prostate cancer specimens by in situ hybridization (ISH) and immunohistochemical analyses, including high-grade prostatic intraepithelial neoplasia specimens, primary androgen-dependent tumors specimens, hormone-refractory tumors specimens, and bone metastases specimens[4]. Moreover, Han et al. showed that the Gleason score, tumor stage, seminal vesicle invasion, and distant metastases, as well as increased risk of biochemical recurrence or progression to androgen-independent disease, were all closely correlated to the increased PSCA expression [5]. Consistent with these findings, our previous study also reported the positive correlation between the PSCA expression level and the increased pathological grade (poor cell differentiation), heightened clinical stage, and androgen independence [6, 7].

As to the intervention means of prostate cancer, androgen receptor (AR) targeting is an important therapeutic strategy and the next-generation androgen receptor (AR) pathway inhibitors (ARPIs) prolong the survival of patients [8]. However, androgen deprivation therapy (ADT) is effective only in the initial stage of the treatment, and most tumors progress to castration-resistant prostate cancer (CRPC), as well as develop into the neuroendocrine prostate cancer (NEPC) under the following therapeutic process [9]. NEPC does not secrete prostate-specific antigen (PSA) and is a highly aggressive subtype of prostate cancer characterized by the following features: unresponsiveness to hormone therapy and expression of neuroendocrine markers such as enolase 2 (NSE) [10, 11]. The prognoses of patients with NEPC are always awful due to the difficulties that exist in the diagnosis and treatment. Jemal et al. reported that approximately 25% of the nearly 34,000 cases of lethal prostate cancer per year is caused by the NEPC in the United States [12]. In addition, some studies indicated that PSCA may be a potential treatment target of gene therapy, when the prostate cancer becomes refractory to the hormone therapy [13, 14].

However, little is known about the correlation between the PSCA and NSE expressions in patients with prostate cancer. Therefore, we investigated the relationship between the expressions of PSCA and NSE in prostate cancer in the present study. We also explored their correlation with clinicopathological features and their prognostic value for patient survival.

## 2. Materials and Methods

### 2.1. Tissue Samples and Clinical Data

Formalin-fixed and paraffin-embedded consecutive sections from 118 patients who received Transurethral Resection of the Prostate (TURP) (*n* = 47) or prostatectomy (*n* = 71) from 2009 to 2019 at our hospital were used in the study. Seventy-eight patients were part of a previous study [15]; however, IHC analyses for those cases were remade and scored with the entire cohort. Patients ranged in age from 42 to 87 years; the mean age was 71.10 ± 9.60 years old. Other clinical data, including the Gleason score, preoperative prostate-specific antigen (PSA) level, tumor stage, follow-up status, and surgical intervention, were retrospectively obtained from our hospital. All patients were histopathologically diagnosed with prostate cancer according to their prostate tissue specimens. All patients signed written informed consent forms before taking part in this study, and the study protocol was approved by the Ethics Committee of the First Affiliated Hospital of Guangzhou Medical University.

### 2.2. Immunohistochemical (IHC) Staining

Two sections from the same patient were immunostained with rabbit polyclonal anti-PSCA antibody (1 : 50, #ab64919, Abcam, Cambridge, UK) and rabbit polyclonal anti-NSE antibody (1 : 100, #ab79757, Abcam, Cambridge, UK).

### 2.3. Evaluation of PSCA and NSE Protein Expressions

PSCA protein expression was mainly localized in the cell membrane, but NSE protein expression also was localized in the cytoplasm except for the cell membrane. Both of the two proteins present brownish yellow color and granular shape after IHC. And the staining of PSCA and NSE was independently evaluated by two experienced pathologists using a semiquantitative scale based on the staining intensity and extent. The score of IHC staining intensity of each microscopic field ranges from 0 to 3: 0 means that the staining is negative, score 1 represents weak positive staining, score 2 represents moderate positive staining, and score 3 represents strong positive staining. Besides, the IHC extent of staining of each microscopic field was scored from 0 to 4 on the basis of the percentage of positive staining in the section: 0% positive staining scored 0, 1%–25% positive staining scored 1, 26%–50% positive staining scored 2, 51%–75% positive staining scored 3, and >75% positive staining scored 4. The results of staining intensity multiplied by the percentage of positive staining were the final score of each microscopic field. Four visual fields of each slice were randomly selected for the IHC score, and the average score of the four visual fields was the final score of the slice. The expressions of the two proteins were compared in the same visual field of two different sections. And the final score of each specimen was graded, 0 to 1 as 0 grade, 2 to 4 as 1 grade, 5 to 8 as 2 grade, and 9 to 12 as 3 grade [16]. The final scores of all specimens were used for statistical analysis.

### 2.4. Statistical Analysis

A Spearman rank test was used to analyze the correlation between PSCA and NSE. The score data of IHC were presented as mean ± standard deviation ($\bar{\text{x}}\pm \text{SD}$). Differences in PSCA and NSE scores within each group were analyzed by comparing the mean value using the Student *t*-test and one-way ANOVA analysis when indicated. The correlation between two indices was analyzed by Spearman correlation. Overall survival (OS) was defined from the date of first diagnosis to the date of death or last follow-up. The overall survival (OS) curve was estimated using the Kaplan-Meier method, and the differences in the survival curves were compared using the log-rank test. Univariate and multivariate analyses were performed using Cox's regression model, in which the proportional hazard assumption was tested by the Schoenfeld residual test. SPSS 22.0 software (SPSS Inc., Chicago, IL, USA) was used for statistical analysis in this study. *P* < 0.05 was considered indicative of statistical significance.

## 3. Results

### 3.1. Clinicopathological Characteristics of Patients

The mean age of the patients was 71.10 ± 9.60 years (median, 73 years; range, 42-87years). Of the 118 patients, 50 (42.4%) were less than or equal to 70 years old and 68 (57.6%) were older than 70 years old. 28.8% of the patients had a preoperative PSA level less than 10 ng/ml, 16.9% of the patients had a preoperative PSA level from 10 to 20 ng/ml, and 54.3% had a preoperative PSA greater than 20 ng/ml. The Gleason scores were stratified into Gleasons 2 to 6 in 33 patients (28.0%), Gleason 7 in 30 (25.4%), and Gleasons 8 to 10 in 55 patients (46.6%). The pathologic T (pT) stage grouping was ≤T2 in 71 patients (60.1%) and >T2 in 18 (39.9%). Of all the patients, 43 (36.4%) presented metastasis, 75 (63.6%) were absent according to our definition that any lymph node metastasis and distant metastasis were concluded as metastasis. There were 31 patients who had received endocrine therapy, including adjuvant therapy, neoadjuvant therapy, or simple endocrine therapy, while 87 patients had not received endocrine therapy. In addition, 61 patients (51.7%) survived, 25 patients (21.2%) died, and 32 patients (27.1%) were lost to follow-up. 71 patients received prostatectomy, and 47 patients received TURP (Table 1).

### 3.2. Immunohistochemical Assay and the Correlation of PSCA Expression with Clinicopathological Factors

**Figure 1:**
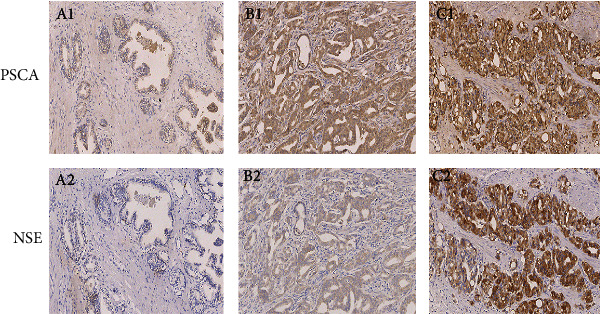
PSCA and NSE protein expression in different patients' prostate cancer lesions using immunohistochemistry. (A1) (IHC score: 1; IHC grade: 0) and (A2) (IHC score: 0; IHC grade: 0) are from the same patient's prostate cancer tissue; (B1) (IHC score: 9; IHC grade: 3) and (B2) (IHC score: 6; IHC grade: 2) are from the same patient's prostate cancer tissue; (C1) (IHC score: 7; IHC grade: 2) and (C2) (IHC score: 6; IHC grade: 2) are from the same patient's prostate cancer tissue. Abbreviations: PSCA: prostate stem cell antigen; NSE: enolase 2.

**Figure 2:**
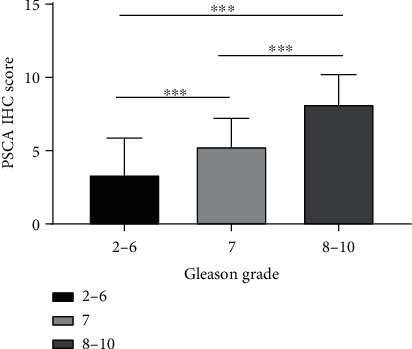
Distribution of PSCA IHC score according to Gleason grade. Abbreviation: PSCA: prostate stem cell antigen.^∗∗∗^*P* < 0.001.

The immunohistochemical expression of PSCA with different semiquantitative IHC scores is shown in Figure 1. The mean (±SD) IHC score of PSCA was 6.11 ± 2.94 (range: 0.00–12.00) in all patients. The PSCA IHC scores in the patients with higher Gleason scores were significantly higher than those with lower Gleason scores (Table 2 and Figure 2). However, the difference of score between different age groups was not significant statistically (*P* = 0.131). As to the pathologic T (pT) stage, the IHC scores of PSCA of the patients in >T2 stages were statistically higher compared to those in ≤T2 stages (Table 2). The same significant difference was also observed within the metastasis groups (*P* < 0.001). Nevertheless, no significant correlation between the PSCA IHC scores and the hormone treatment was detected (Table 2).

### 3.3. NSE Immunohistochemical Expression and Its Correlation with Clinicopathological Factors

**Figure 3:**
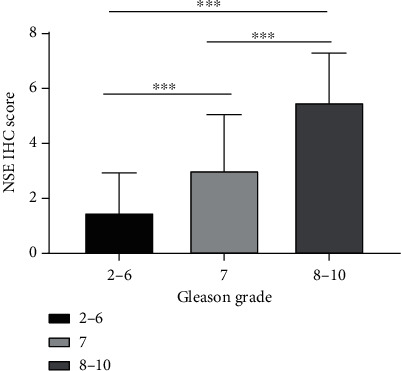
Distribution of PSCA IHC score according to Gleason grade. Abbreviation: NSE: enolase 2. ^∗∗∗^*P* < 0.001.

A different immunohistochemical expression of NSE in different specimens is presented in Figure 1. The mean (±SD) IHC score of NSE was 3.75 ± 2.46, ranged from 0 to 9, in all subjects. NSE expression in elderly patients was a little higher than in those with a relatively young age (3.87 ± 2.48 versus 3.59 ± 2.45, respectively), but the difference was not significant (*P* = 0.545). Nevertheless, the patients with higher Gleason scores had significantly higher IHC scores of NSE compared to those with lower Gleason scores (Table 3 and Figure 3). From the viewpoint of pT stage, the difference of the NSE IHC score between the ≤T2 stage group and the >T2 stage group was significantly observed (3.17 ± 2.33 versus 4.63 ± 2.41, respectively; *P* = 0.001). Besides, the significant correlations between the NSE IHC scores and metastasis were found (Table 3).

### 3.4. Correlations between Immunohistochemical Markers and Preoperative PSA Level of Patients

In the point of general preoperative PSA level of all patients, a significant positive correlation between PSCA and NSE was explored (*r* = 0.641, *P* < 0.001) as shown in Table 4. No statistically significant relationship was found between the preoperative PSA level and PSCA or between the preoperative PSA level and NSE (*r* = 0.087, *P* = 0.348; *r* = 0.055, *P* = 0.552, respectively).

However, a significantly negative correlation can be noted not only between the preoperative PSA level and PSCA (*r* = −0.361, *P* = 0.036) but also between the preoperative PSA level and NSE (*r* = −0.372, *P* = 0.030) when these correlations were detected in patients with preoperative PSA < 10 ng/ml. In addition, the significant positive correlation between PSCA and NSE still can be observed in the above-mentioned patients (Table 4).

In the patients with hormone therapy, a significant positive relationship was noted between PSCA and NSE (*r* = 0.587, *P* = 0.001). But PSCA or NSE was not associated with the preoperative PSA level in these patients (*r* = 0.180, *P* = 0.333; *r* = 0.201, *P* = 0.279, respectively).

### 3.5. Survival Analysis

**Figure 4:**
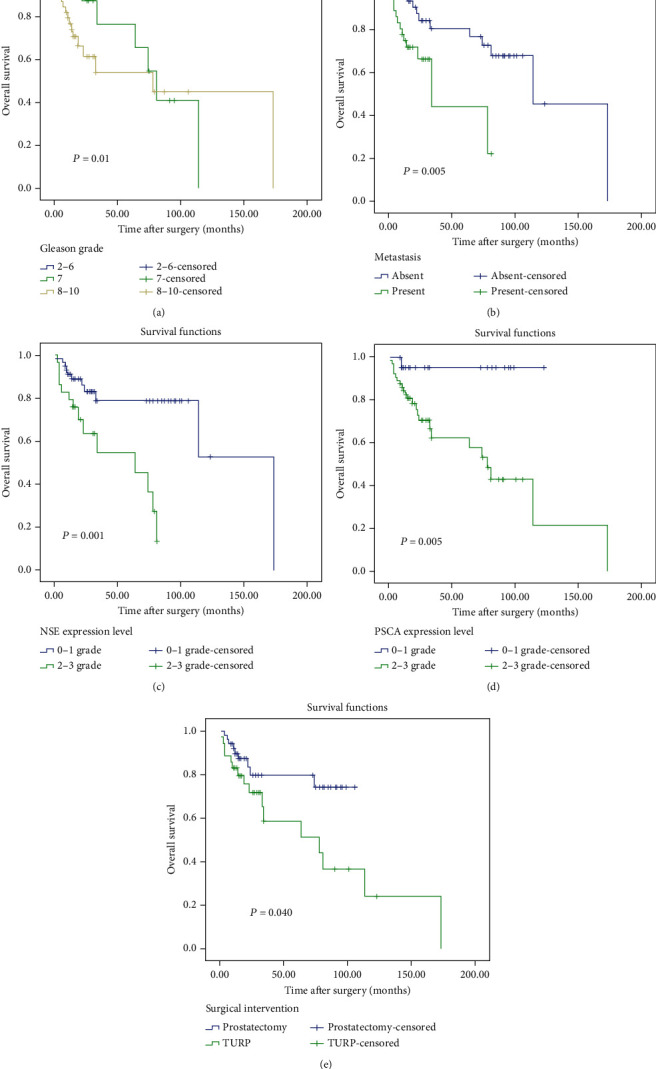
Kaplan-Meier plots for overall survival in patients with prostate cancer according to different clinicopathological factors. (a) Gleason grade; (b) metastasis; (c) PSCA expression level; (d) NSE expression level; (e) Surgical intervention. Abbreviations: PSCA: prostate stem cell antigen; NSE: enolase 2. Significant *P* values were determined using the log-rank test.

Survival analysis was utilized to evaluate the relationship between the PSCA or NSE expression and prostate cancer prognosis. The duration of the follow-up ranged from 1 to 173 months, and the median survival time was 21 months. The OS rate was 70.9%. Gleason score (*P* = 0.01), surgical intervention (*P* = 0.040), and metastasis (*P* = 0.005) were the single prognostic factors. Besides, Kaplan-Meier analysis also showed that patients with a higher grade of PSCA and NSE expressions had significantly poorer survival than those with lower expressions of these markers in prostate cancer patients (*P* = 0.005 and *P* = 0.001, respectively; Figure 4). In contrast, univariate analysis using Kaplan-Meier showed that the OS rate was not significantly influenced by patient age (*P* = 0.531), preoperative PSA level (*P* = 0.558), pT stage (*P* = 0.304), and hormone therapy status (*P* = 0.09). Univariate and multivariate analyses were utilized to evaluate whether the PSCA or NSE expression level and various clinicopathological features were independent prognostic parameters of prostate cancer patient outcomes. In the univariate model, significant associations were detected between OS and Gleason score (hazard ratio [HR] = 2.313; *P* = 0.004), metastasis status (HR = 3.363; *P* = 0.007), PSCA expression level (HR = 10.068; *P* = 0.024), NSE expression level (HR = 3.841; *P* = 0.002), and surgical intervention (HR = 2.346; *P* = 0.046). However, no clinicopathological factor continued to be a significant predictor of OS in multivariate analysis (Table 5).

## 4. Discussion

The diagnosis and treatment of prostate cancer are different challenges for urologists and pathologists [12]. Some clinicopathological factors like Gleason grade, PSA level, clinical stage, or pathological stage were always used to assess the prognosis of the cancer. But the instability and susceptibility of these above factors still exist during the management of the disease [8, 17–19]. Therefore, new biomarkers are needed.

Some studies have indicated that PSCA was involved in the progression, metastasis, and development of some cancers, including gastric cancer [20], bladder cancer [21], clear cell renal cell carcinoma [22], breast cancer [23], pancreatic cancer [24, 25], gallbladder cancer [26, 27], and esophageal squamous cell carcinoma [28] in recent years. As to the prostate, Zhang et al. found that Chinese patients with the rs1045531 AC genotype of PSCA have a higher risk in suffering prostate cancer when undergoing prostate biopsy [29]. The data from Taeb et al. show that expression of PSCA increased from benign prostate tissues (BPH) and HGPIN to prostate cancer and that its expression in prostate cancer was positively associated with poor cell differentiation in Iran [30]. Similar to these results, our previous studies also revealed that PSCA can promote prostate cancer proliferation and cell-cycle progression and can increase the metastatic potentials by EMT in prostate cell lines [15, 31].

In the present study, patients with higher Gleason score and progressive pT stage had a higher PSCA IHC score, and the difference among groups was statistically significant.

The result is consistent with the study that reported that the level of PSCA expression was increased with a higher Gleason score of prostate cancer [30, 32]. Moreover, the results in the current study that the PSCA IHC scores of prostate cancer specimens were higher in patients with metastasis also support the previous findings by Garcia-Hernandez et al. In their research, PSCA was a key role in the tumor microenvironment inducing tumor growth, metastasis, and drug resistance [33]. Therefore, the expression of the prostate stem cell antigen can be an assistant measure for the clinical diagnosis and progressivity assessment of prostate cancer.

In some former studies, upregulation of PSCA in prostate cancer was significantly correlated with progression to androgen independence, biochemical recurrence, and/or distant metastases [4, 5, 34]. Interestingly, the neuroendocrine prostate cancer (NEPC) is significantly associated with androgen independence, susceptibility to distant metastasis, and insensitivity to all forms of hormonal treatment [35–38]. Besides, NEPC is characterized by positive immunohistochemical (IHC) staining for CHGA, SYP, and neuron-specific enolase (NSE) [39]. Therefore, detection of the relationship between the PSCA and NSE expressions in prostate cancer is necessary. Similar to the expression of PSCA in the prostate cancer specimens, intensive IHC expression of NSE was observed in patients with a higher Gleason score, advanced pT stage, and metastasis in the present study, in agreement with the findings from previous studies [40, 41].

Moreover, a significant positive correlation was noted between the PSCA and NSE expressions in all prostate cancer cases in the present study, but not found between PSA and PSCA or NSE. However, the significant negative relationship between PSA and PSCA or NSE was detected in patients with preoperative PSA < 10 ng/ml. This finding may be caused by the low expression of PSA in NEPC [10] and indicate that the prostate stem cell antigen may be involved in the process from prostate cancer to NEPC.

Meanwhile, consistent with other studies [5, 6], a high Gleason grade and the presence of metastasis were associated with shorter OS in the current study. And the surgical intervention can impact the OS of the prostate cancer patient, which may be due to factors such as radical resection of the tumor and expanded lymph node dissection in prostatectomy [18]. Shorter OS was also found in patients with intensive expression of PSCA. As shown in some previous studies, PSCA can promote prostate cancer cell proliferation and escalate the distant metastasis of prostate cancer [15, 31]. Therefore, PSCA may play a carcinogenesis role in the progression of prostate cancer, and its expression level in prostate cancer sections might be a useful parameter for judging the prognosis of patients with prostate cancer. The same relationship between OS and the expression level of NSE was detected, which was consistent with the result by Pascale et al. [42]. Thus, the overexpression of NSE could be considered as a symbol of a poor prognosis and can be a useful biomarker to assess tumor aggressiveness. Nevertheless, none of the five factors was a significant prognostic factor of OS in multivariate analysis; they can be regarded as independent prognostic factors.

## 5. Conclusion

PSCA and NSE expressions correlated with the Gleason score, pT stage, metastasis and poor OS in patients with prostate cancer. Both markers can be used to facilitate the assessment of diagnosis and prognosis in patients with prostate cancer. There is a positive relationship between PSCA and NSE but a negative relationship between PSA and PSCA or between PSA and NSE, and PSCA may play a key role in the progression from prostate cancer to NEPC as an oncogene. These findings indicated that the potential molecular mechanism inducing NEPC behind the PSCA could let it be a promising therapeutic molecular target for NEPC.

## Figures and Tables

**Table 1:** Clinicopathological parameters for patients (*n* = 118).

Clinicopathological parameter	*n* (%)
Age (years)	
≤70	50 (42.4%)
>70	68 (57.6%)
Preoperative PSA level (ng/ml)	
<10	34 (28.8%)
10-20	20 (16.9%)
>10	64 (54.3%)
Gleason score	
2-6	33 (28.0%)
7	30 (25.4%)
8-10	55 (46.6%)
pT stage	
≤T2	71 (60.1%)
>T2	47 (39.9%)
Metastasis	
Absent	75 (63.6%)
Present	43 (36.4%)
Hormone therapy	
No	87 (73.7%)
Yes	31 (26.3%)
Follow-up	
Alive	61 (51.7%)
Dead	25 (21.2%)
Loss	32 (27.1%)
Surgical intervention	
Prostatectomy	71 (60.2%)
TURP	47 (39.8%)

Abbreviations: pT stage: pathologic tumor stage; PSA: prostate-specific antigen; TURP: Transurethral Resection of the Prostate.

**Table 2:** PSCA IHC expression and its correlation with clinicopathological factors (*n* = 118).

Factors	Number of patients	Score of IHC expression^a^	*P* value
Age (years)			
≤70	50	5.63 ± 3.05	0.131^b^
>70	68	6.46 ± 2.84	
Gleason grade			
2-6	33	3.39 ± 2.47	<0.001^c^
7	30	5.31 ± 1.90	
8-10	55	8.19 ± 2.01	
pT stage			
≤T2	71	5.43 ± 2.93	0.002^b^
>T2	47	7.12 ± 2.69	
Hormone therapy			
No	87	6.43 ± 3.02	0.046^b^
Yes	31	5.20 ± 2.56	
Metastasis			
Absent	75	5.37 ± 3.01	<0.001^b^
Present	43	7.40 ± 2.34	

Abbreviations: PSCA: prostate stem cell antigen; pT stage: pathologic tumor stage. ^a^Mean ± SD; ^b^Student *t*-test; ^c^one-way ANOVA analysis.

**Table 3:** NSE IHC expression and its correlation with clinicopathological factors (*n* = 118).

Factors	Number of patients	Score of IHC expression^a^	*P* value
Age (years)			
≤70	50	3.59 ± 2.45	0.545^b^
>70	68	3.87 ± 2.48	
Gleason grade			
2-6	33	1.50 ± 1.44	<0.001^c^
7	30	3.02 ± 2.02	
8-10	55	5.51 ± 1.79	
pT stage			
≤T2	71	3.17 ± 2.33	0.001^b^
>T2	47	4.63 ± 2.41	
Hormone therapy			
No	87	3.97 ± 2.59	0.101^b^
Yes	31	3.12 ± 1.95	
Metastasis			
Absent	75	3.18 ± 2.48	0.001^b^
Present	43	4.73 ± 2.12	

Abbreviations: NSE: enolase 2; pT stage: pathologic tumor stage. ^a^Mean ± SD; ^b^Student *t*-test; ^c^one-way ANOVA analysis.

**Table 4:** Correlation among immunohistochemical markers in clinicopathological factors.

Marker expression	PSA	PSCA	NSE
*General preoperative PSA level*			
PSA			
*r*	NA	0.087	0.055
*P* value	NA	0.348	0.552
PSCA			
*r*	0.087	NA	0.641^a^
*P* value	0.348	NA	<0.001
NSE			
*r*	0.055	0.64^a^	NA
*P* value	0.552	<0.001	NA
*PreoperativePSA* < 10 *ng*/*ml*			
PSA			
*r*	NA	-0.361^a^	-0.372^a^
*P* value	NA	0.036	0.030
PSCA			
*r*	-0.361^a^	NA	0.685^a^
*P* value	0.036	NA	<0.001
NSE			
*r*	-0.372^a^	0.685^a^	NA
*P* value	0.030	<0.001	NA
*Patients with hormone therapy*			
PSA			
*r*	NA	0.180	0.201
*P* value	NA	0.333	0.279
PSCA			
*r*	0.180	NA	0.587^a^
*P* value	0.333	NA	0.001
NSE			
*r*	0.201	0.587^a^	NA
*P* value	0.279	0.001	NA

Abbreviations: PSA: prostate-specific antigen; PSCA: prostate stem cell antigen; NSE: enolase 2. ^a^Correlation is significant at the *P* = 0.05 level (test of significance, Spearman correlation).

**Table 5:** Univariate and multivariate Cox logistic regression analysis of overall survival in patients with prostate cancer.

Clinicopathological features	Univariate			Multivariate		
	HR	95% CI	*P* value	HR	95% CI	*P* value
Age	1.308	0.563-3.039	0.533			
Preoperative PSA level	1.328	0.783-2.253	0.293			
Gleason score	2.313	1.299-4.118	0.004	1.347	0.630-2.878	0.442
pT stage	1.538	0.672-3.520	0.308			
Metastasis status	3.363	1.390-8.134	0.007	1.572	0.564-4.387	0.387
Hormone therapy	0.306	0.072-1.308	0.110			
PSCA expression level	10.068	1.355-74.805	0.024	4.250	0.466-38.734	0.199
NSE expression level	3.841	1.647-8.957	0.002	1.711	0.638-4.584	0.286
Surgical intervention	2.346	1.014-5.428	0.046	1.750	0.730-4.192	0.210

Abbreviations: HR: hazard ratio; CI: confidence interval; pT stage: pathologic tumor stage; PSCA: prostate stem cell antigen; NSE: enolase 2.

## Data Availability

The data used to support the findings of this study are included within the article.
